# Design and characterization of protective pan-ebolavirus and pan-filovirus bispecific antibodies

**DOI:** 10.1371/journal.ppat.1012134

**Published:** 2024-04-11

**Authors:** Ariel S. Wirchnianski, Elisabeth K. Nyakatura, Andrew S. Herbert, Ana I. Kuehne, Shawn A. Abbasi, Catalina Florez, Nadia Storm, Lindsay G. A. McKay, Leandrew Dailey, Erin Kuang, Dafna M. Abelson, Anna Z. Wec, Srinjoy Chakraborti, Frederick W. Holtsberg, Sergey Shulenin, Zachary A. Bornholdt, M. Javad Aman, Anna N. Honko, Anthony Griffiths, John M. Dye, Kartik Chandran, Jonathan R. Lai

**Affiliations:** 1 Department of Biochemistry, Albert Einstein College of Medicine, Bronx, New York, United States of America; 2 Department of Microbiology and Immunology, Albert Einstein College of Medicine, Bronx, New York, New York, United States of America; 3 Virology Division, United States Army Medical Research Institute of Infectious Diseases, Frederick, Maryland, United States of America; 4 The Geneva Foundation, Tacoma, Washington, United States of America; 5 Department of Virology, Immunology, and Microbiology; and National Emerging Infectious Diseases Laboratories, Boston University School of Medicine, Boston, Massachusetts, United States of America; 6 Mapp Biopharmaceutical Inc., San Diego, California, United States of America; 7 Integrated BioTherapeutics, Inc., Rockville, Maryland, United States of America; University of Texas Medical Branch / Galveston National Laboratory, UNITED STATES

## Abstract

Monoclonal antibodies (mAbs) are an important class of antiviral therapeutics. MAbs are highly selective, well tolerated, and have long *in vivo* half-life as well as the capacity to induce immune-mediated virus clearance. Their activities can be further enhanced by integration of their variable fragments (Fvs) into bispecific antibodies (bsAbs), affording simultaneous targeting of multiple epitopes to improve potency and breadth and/or to mitigate against viral escape by a single mutation. Here, we explore a bsAb strategy for generation of pan-ebolavirus and pan-filovirus immunotherapeutics. Filoviruses, including Ebola virus (EBOV), Sudan virus (SUDV), and Marburg virus (MARV), cause severe hemorrhagic fever. Although there are two FDA-approved mAb therapies for EBOV infection, these do not extend to other filoviruses. Here, we combine Fvs from broad ebolavirus mAbs to generate novel pan-ebolavirus bsAbs that are potently neutralizing, confer protection in mice, and are resistant to viral escape. Moreover, we combine Fvs from pan-ebolavirus mAbs with those of protective MARV mAbs to generate pan-filovirus protective bsAbs. These results provide guidelines for broad antiviral bsAb design and generate new immunotherapeutic candidates.

## Author summary

Filoviruses, such as Ebola virus and Marburg virus (EBOV and MARV, respectively), cause severe hemorrhagic fever with a high mortality rate in humans. Monoclonal antibodies (mAbs) are effective treatments for filovirus infection, but current therapies have limited breadth. Furthermore, a single mAb is susceptible to development of resistance. Here, we used protein engineering to create “bispecific” antibodies in which activities of two different mAbs were combined into one. These bispecific antibodies had broad activity, in one case providing protection against lethal challenge by two distant clades of filoviruses (EBOV and MARV). The bispecific antibodies were also less susceptible to resistance mutations. This work provides a roadmap for development of new bispecific antibody therapies for filoviruses.

## Introduction

Filoviruses are negative-strand RNA viruses that cause severe hemorrhagic fever with mortality rates of ~30–90%. Filoviruses are classified into six genera, but nearly all human disease has been caused by three ebolaviruses (Ebola virus, EBOV, Sudan virus, SUDV, and Bundibugyo virus, BDBV) and two marburgviruses (Marburg virus, MARV, and Ravn virus, RAVV) [1]. The 2013–2016 EBOV epidemic illustrated the capacity for widespread dissemination of these viruses in urban settings, despite their requirements for direct contact with infected mucosal surfaces for human-to-human transmission [2]. The epidemic affected nine countries, with the highest numbers of cases and deaths in Guinea, Liberia, and Sierra Leone. Overall, there were over 28,000 suspected cases and 11,325 deaths [3]. All other filovirus outbreaks have been much smaller in comparison, but the potential for virulent filoviruses to emerge is a significant concern. For example, SUDV caused a 164-case outbreak (77 deaths) in Uganda in late 2022 [4]. Thus, there is an urgent need for development of new, broadly active filovirus countermeasures.

Monoclonal antibodies (mAbs) are a promising therapeutic modality for filoviruses and other viral pathogens [5–11]. MAbs are generally well-tolerated with few off-target effects, have long in vivo half-life, and—especially important for viral diseases—the capacity to recruit immune mediators and clear infected cells via their Fc region. MAb therapies have been approved for treatment of EBOV, SARS-CoV2, and respiratory syncytial virus (RSV), and are under advanced development for other viral diseases. Inmazeb consists of a cocktail of three EBOV mAbs, and Ebanga is a single-component therapy [7,8]. Other advanced mAb filovirus therapies include the broad-spectrum two-component MBP134 cocktail, which has been demonstrated to protect non-human primates from lethal challenge by EBOV, BDBV, and SUDV, and MBP091, a MARV- and RAVV-specific monotherapy [12–14].

The filovirus glycoprotein (GP) is required for cellular entry and is the target for all filovirus mAb therapies [15–18]. Prefusion GP is a trimer comprising two subunits—the surface subunit GP1, which contains the receptor-binding site (RBS), and the transmembrane subunit GP2, which mediates viral membrane fusion. Infection is initiated by viral attachment, followed by internalization of virions and delivery to late endosomal/lysosomal compartments where host cysteine proteases remove the mucin-like domain and glycan cap of GP1 (“cleaved GP”, or “GP_CL_”). These cleavages expose the highly conserved RBS, which binds to the viral receptor Niemann-Pick C-1 (NPC1). GP-NPC1 binding, together with other incompletely defined stimuli, triggers conformational changes in GP2 that lead ultimately to fusion of the host and viral membranes [19–22]. The overall prefusion structure of conserved elements in GP is similar for EBOV, SUDV, and MARV, but the location of the mucin-like domain (MLD) differs between ebolaviruses and MARV [23,24]. The EBOV prefusion GP adopts a “chalice” structure with the MLD projecting outwards from the viral particle extending from each of the protomers and blocking the RBS. In contrast, the MLD is located more equatorially in MARV prefusion GP, exposing the RBS but blocking the “base” of the chalice [23]. A C–terminal portion of the MARV MLD is located in the mature GP2 subunit, where it forms an N–terminal “wing” domain [25].

Sites of susceptibility for viral neutralization, a key correlate of protection for mAbs in filoviruses, also appear to differ between ebolaviruses and marburgviruses [23]. In EBOV, the “base” of GP is engaged by both monospecific as well as broadly neutralizing mAbs. For example, KZ52 (EBOV) and 16F6 (SUDV) are two monospecific mAbs that bind to the base, but mAbs that engage more of the GP2 fusion loop, such as ADI-15878 (one component of the MBP134 cocktail) tend to have broader neutralization profiles [12,13,15,17,24,26–28]. For MARV, MR191 and MR72 both engage the RBS and weakly neutralize GP. These mAbs also recognize the RBS in EBOV GP—a testament to its highly conserved nature among filoviruses—but only after the RBS is exposed by GP cleavage. MR191 and MR72 neutralize both viruses much more potently after GP cleavage, concordant with increased RBS exposure. [23,29,30]. Another important component of mAb-mediated protection against filoviruses is Fc effector function [18,31]. Although many filovirus mAbs can exhibit Fc activity, a number of mAbs that are correlated with protection target the glycan cap [18,31].

MAb therapies that target two or more epitopes are advantageous for viral immunotherapy, because they are less susceptible to viral escape by a single mutation. Such multi-epitope targeting can be accomplished either by mixing canonical mAbs together into cocktails (e.g., Inmazeb or MBP134) or by physically joining variable domains (Fvs) from two antibodies as bispecific (bsAbs) or multispecific (e.g., trispecific) antibodies [32,33]. We have previously reported the development of bsAbs against filoviruses, and Crimean-Congo hemorrhagic fever virus (CCHFV), and other groups have targeted SARS-CoV-2 and HIV-1 [32–41]. BsAbs provide the capacity to engage two epitopes, but within a single molecule. Here, we utilize this approach to develop broad bsAb therapies against filoviruses. We identified multiple bsAbs with pan-ebolavirus neutralizing activity and several bsAbs with pan-filovirus protective activity in mice.

## Results

### Bispecific antibody design

We designed three groups of bsAbs that were predicted to function via distinct mechanisms. In Group I bsAbs, variable domains (Fvs) from broadly neutralizing mAbs were combined in a variety of formats (discussed below). The hypothesis behind Group I bsAbs was that engagement of multiple epitopes simultaneously on the viral surface would provide the potential for synergistic neutralization, as well as possibly mitigate risk of viral escape via a single point mutation in GP. The specific combination of mAbs ADI-15878 and ADI-23774 comprise the broadly neutralizing MBP134 cocktail that protects against lethal challenge in rodent and nonhuman primate models [12,13,28]. MBP134 has completed phase I clinical trials and was deployed in an emergency setting during the 2022 SUDV outbreak in Uganda [42]. ADI-15878 binds a conserved epitope near the fusion loop, and ADI-23774 is an affinity matured version of parental mAb ADI-15946 which binds a non-competing epitope in the GP prefusion base [12,13,26,28]. Both ADI-15878 and ADI-23774 separately neutralize multiple ebolaviruses in vitro and protect against lethal EBOV challenge in mice. The MBP134 cocktail consists of an equimolar mixture of the two mAbs. Thus, one set of bsAbs in Group I combines Fvs from ADI-15878 (abbreviated “A878” herein) and ADI-23774 (“A774”) into a single agent.

**Fig 1:**
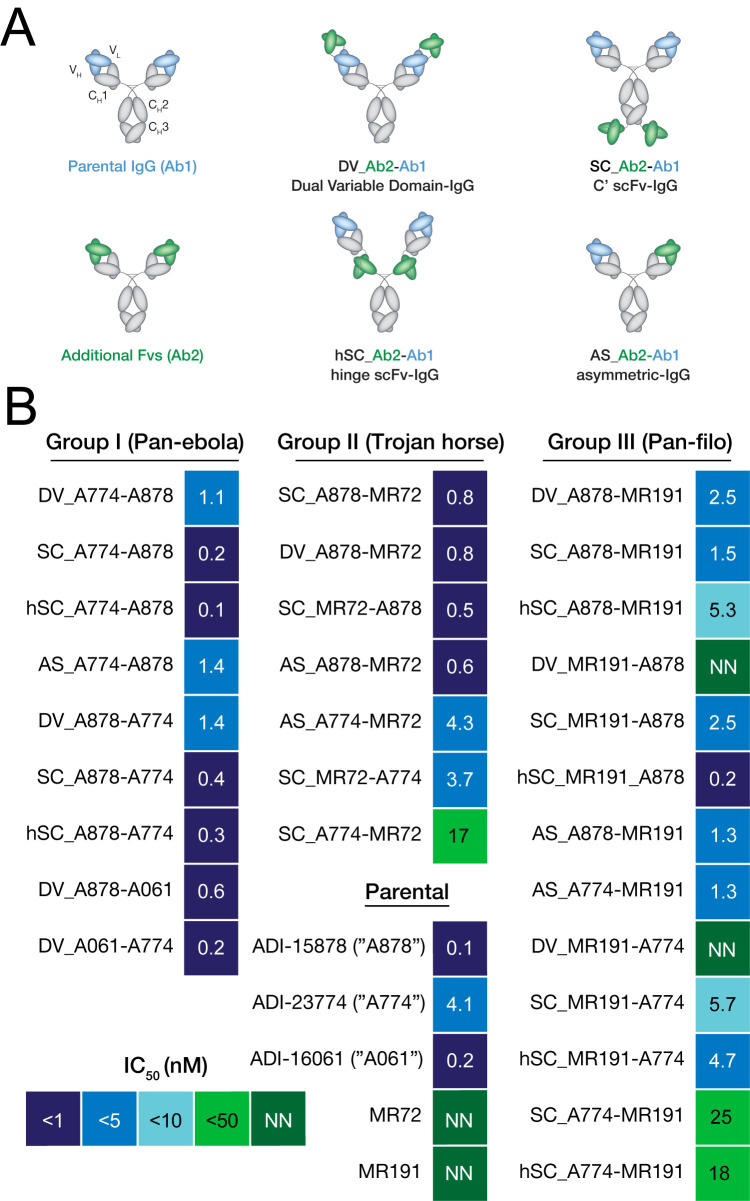
Schematics of bsAbs and neutralization activity against rVSV-EBOV. (A) Schematic of bsAbs constructs, along with their nomenclature. The first designation in bsAb names reflects their format (“DV” for DVD-Ig, “SC” for scFv-IgG, “hSC” for hinge scFv-IgG, and “AS” for asymmetric). For symmetric bsAbs, Fvs with blue domains are the ones remaining in Fab format and their name appears second in order. For example, “DV_A774-A878” is a DVD-Ig format with A774 Fvs as the green (outer) domains and A878 Fvs as the blue (inner). (B) Heat map of neutralizing half-maximal inhibitory concentration (IC_50_) values against rVSV-EBOV for the antibody panel. mAbs or bsAbs with curves that did not cross the 50% threshold are designated as non-neutralizing (NN).

We have previously described antiviral bsAbs employing the Dual Variable Domain-Ig format (DVD-Ig) [43], the single chain Fv-IgG fusion at the heavy chain C-terminus (scFv-IgG), as well as the asymmetric Duobody format [44,45]. The latter design (Duobody) is generated by expressing each “half” of the asymmetric bsAb separately and then combining them via “controlled Fab exchange” under mildly reducing conditions to result in an IgG1-like molecule with one arm for each Fv specificity [45]. We evaluated all three formats for A878/A774 bsAbs in addition to the BiS4 format that incorporates a scFv in the hinge of an IgG1 (hSC-Ig, [46]) (**Fig 1A**). For the DVD-Ig molecules, two Fv orientations are possible: one with A878 as the “parental, inner” domains and A774 as the “outer” domains, and vice versa. For scFv-IgGs, Fvs from either mAb could be appended as a scFv to a parental IgG. We previously reported in other systems that differential arrangements of Fvs within these scaffolds can have strong effects on activity that are not straightforward to predict *a priori* [34]. Accordingly, for both DVD-Ig and scFv-IgG bsAbs, we generated versions in which the parental IgG1 molecule bore the A878 Fvs, and then another set based on the A774 parental IgG1 (**Fig 1** and **S1 Table**). Other Group I bsAbs were similar in design but combined the Fvs of A878 or A774 with those of ADI-16061 (“A061”), a GP2 stalk-binding antibody with neutralizing activity against EBOV and BDBV [28]. For this set of bsAbs, however, we only tested the tetravalent DVD-Ig and scFv-IgG formats. The nomenclature scheme used for bsAbs is as follows: each bsAb begins with a designation for format (“DV” for DVD-Ig, “SC” for scFv-IgG, “hSC” for hinge-scFv a.k.a. BiS4, and “AS” for asymmetric a.k.a. Duobody); subsequently, the names of the two parental mAbs are listed, with the second parental mAb being the one whose Fvs remain as an Fab in the symmetric constructs (see **Fig 1A** color scheme).

Group II bsAbs were designed with a “Trojan Horse” mechanism in mind [36], in which one set of Fvs could act as a shuttle for endosomal delivery of a second set of Fvs that block the critical NPC1-GP_CL_ interaction. We previously combined Fvs from human mAb MR72, which targets the RBS, with Fvs from mAbs that target broadly reactive, but non-neutralizing epitopes on prefusion GP [36]. We demonstrated that such Trojan Horse bsAbs were neutralizing and protective via a mechanism in which the non-neutralizing Fvs exploited viral particles themselves for delivery to endosomal compartments where the MR72 Fvs could block NPC1 binding and thus prevent membrane fusion. More recently, we have demonstrated that endosomal delivery of MR72 Fvs for neutralization could also be achieved by targeting the cell-surface host cation-independent mannose-6-phosphate receptor [37]. For Group II bsAbs, we reasoned that the neutralizing activity of Trojan Horse bsAbs could potentially be augmented by combining Fvs targeting both RBS and prefusion epitopes in GP. To this end, the Fvs from MR72 were combined with those of A878, or A774 in both DVD-Ig and scFv-IgG formats (**Fig 1** and **S1 Table**).

Group III bsAbs explored broadening bsAb antiviral breadth beyond ebolaviruses to include activity against marburgviruses by combining Fvs for A878 or A774 with the marburgvirus mAb, MR191, which was found to be protective against lethal disease in non-human primates [14,47]. Although the core three-dimensional structures of GP_CL_ are similar for EBOV and MARV, differential furin processing ultimately results in different spatial locations of the mucin-like domain (MLD). In EBOV, the MLD is located higher on the “chalice”, projecting outwards from the glycan cap and blocking any potential interactions between mAbs and the RBS. However, in MARV, the mucin-like domain is located equatorially on the spike as the GP2 wing domain, blocking the base epitopes but leaving the RBS partially exposed [23,47]. Thus, we envisioned that the only possibility for achieving pan-filovirus bsAbs would be to combine Fvs for broad ebolavirus-specific base binding mAbs with those from RBS-binding MARV mAbs.

Genes for all bsAbs were constructed from synthetic DNA fragments encoding sequences of the parental mAb Fv regions. As previously reported, all bsAbs were expressed in ExpiCHO cells using the two-plasmid pMAZ expression system and purified by protein A chromatography [33–37,48].

### Virus neutralization against rVSV-EBOV by Group I and Group II bsAbs

As a primary evaluation for activity, bsAbs were tested for their capacity to inhibit cell entry by a recombinant vesicular stomatitis virus bearing the envelope glycoprotein of EBOV in place of the native glycoprotein G (rVSV-EBOV). The rVSV-EBOV genome also encodes an enhanced GFP which allows for rapid quantification of infected cells by fluorescence microscopy [49]. Group I hSC-Ig and scFv-Ig bsAbs containing A774 and A878 Fvs (i.e., MBP134 cocktail) maintained potent neutralizing activity with rVSV-EBOV (IC_50_s of 0.2 nM and 0.4 nM for SC_A774-A878 and SC_A878-A774, and 0.1nM and 0.3nM for hSC_A774-A878 and hSC_A878-A774, respectively), on par with that of monospecific A878 (0.1 nM), the more potent of the two parental mAbs (**Fig 1B**). Thus, the orientation of the scFv-Ig and hSC-Ig Fvs does not alter neutralizing activity at least for rVSV-EBOV. The DVD-Ig versions of Group I bsAbs had slightly less potent activity (1.1–1.4 nM), although comparable to each other and generally still similar to that of monospecific A878 (0.1nM). An asymmetric design utilizing the Duobody format (AS_A774-A878) had similar activity to the DVD-Igs (1.4 nM). As previously described, the neutralizing potency of A774 (4.1 nM) was lower than that of A878 and all Group I bsAbs, thereby indicating that the potency of Group I bsAbs likely originates from the A878 Fvs.

Two other Group I bsAb designs bearing the A878 or A774 Fvs linked to A061 Fvs in DVD-Ig format also exhibited potent neutralizing activity (0.6 nM for DV_A878-A061 and 0.2 nM for DV_A061-A774), with DV_A878-A061 having slightly lower potency compared to A061 monospecific (0.2 nM).

**Fig 2:**
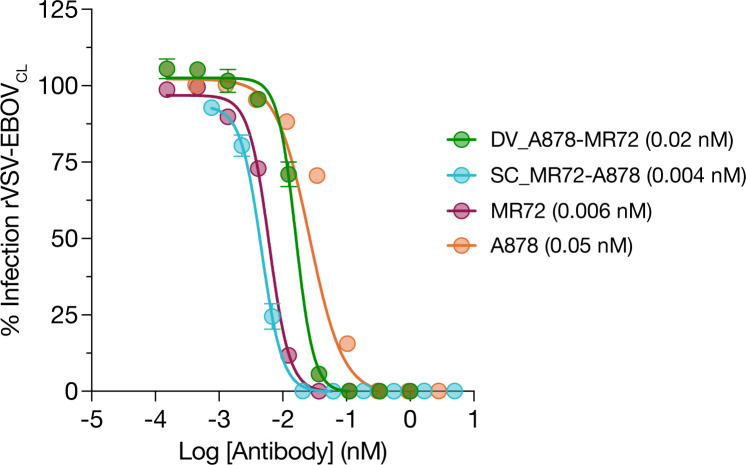
Neutralization of rVSV-EBOV_CL_ by Group II bsAbs. Group II bsAb neutralization activity against rVSV-EBOV_cl_. rVSV-EBOV was pretreated with thermolysin to generate rVSV-EBOVcl prior to incubation with serial dilution of bsAbs. IC_50_ values indicated in the legend in parentheses.

Group II bsAbs that combined Fvs from A878 and MR72 were also similarly potent (DV_A878-MR72 and SC_A878-MR72, both 0.8 nM). However, those designs that incorporated Fvs of A774 coupled to MR72 Fvs were less potent, and in the case of SC_A774-MR72, substantially less so than parental A774 (17 nM vs. 4.1 nM). Given that MR72 binds the RBS, which is not exposed in the prefusion GP and only available for mAb binding upon proteolytic cleavage, we also tested the activity of two Group II mAbs for neutralization against “cleaved” rVSV-EBOV_CL_ generated by treating rVSV-EBOV with thermolysin to mimic endosomal cysteine cathepsin cleavage. These mAbs were highly potent (IC_50_ < 0.1 nM), indicating that the MR72 Fvs maintain activity in these designs (**Fig 2**). As expected, MR72 as a parental mAb had no activity against rVSV-EBOV (**Fig 1B**).

### Breath of virus neutralization by Group I bsAbs

**Fig 3:**
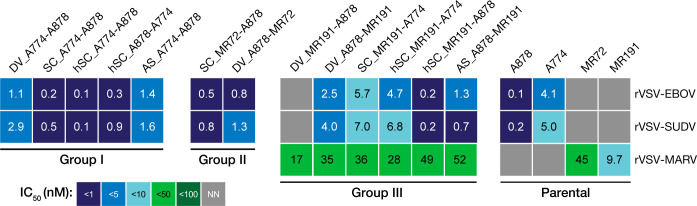
bsAbs exhibit neutralization breadth across rVSV-EBOV, -SUDV, -MARV. Heat map of neutralizing half-maximal inhibitory concentration (IC_50_) values of the bsAb panel against VSV-EBOV, -SUDV, and -MARV. mAbs or bsAbs with curves that did not cross the 50% threshold are designated as non-neutralizers (NN). ND, not determined.

We next explored the capacity of the most potent Group I and II bsAbs to inhibit the infection of rVSVs bearing GP from another disease-causing ebolavirus, SUDV (**Fig 3**). SUDV GP has the lowest homology to EBOV—its envelope glycoprotein amino acid sequence is ~30% divergent [24]. Consequently, only a handful of mAbs, including those contained in the MBP134 cocktail (ADI-15878 and ADI-23774) can cross-neutralize both EBOV and SUDV.

All Group I bsAbs containing the MBP134 Fvs neutralized rVSV-SUDV. However, the potency of DV_A774-A878 was considerably lower than that of SC_A774-A878 (2.9 nM vs. 0.5 nM) or A878 alone (0.2 nM) against this virus. Their SUDV GP-specific activity was closer in magnitude to that of the A774 monospecific control (5.0 nM), suggesting that the neutralizing activity of the A878 may be partially blocked. Asymmetric bsAb AS_A774-A878 was also less potent against rVSV-SUDV (1.6 nM), possibly indicating an advantage to bivalent Fv design, as we have previously found with other “Trojan Horse” bsAb designs [36].

DV_A878-A061 was also less potent than A878 against rVSV-SUDV GP (3.1 nM, **S1 Fig**), possibly again indicating partial blockage of the A878 Fvs in binding to SUDV GP. Notably, however, since activity of A061 itself does not extend to SUDV, any activity observed must result from the A878 variable domains. DV_A061-A774 also was similar in activity against rVSV- SUDV to A774 (2.8 nM, **S1 Fig**). Group II bsAb SC_MR72-A878 maintained A878-like activity against rVSV- SUDV (0.8 nM, respectively), but DV_A878-MR72 had lower potency than the parental mAb (**Fig 2**).

### Pan-filovirus neutralization by Group III bsAbs

The only antibodies known to possess neutralizing activity against EBOV, SUDV, and MARV are engineered bsAbs which target a cell-surface receptor for endosomal trafficking [37]. To explore their pan-filovirus activity, Group III bsAbs were tested against rVSVs bearing EBOV GP or MARV GP [50]. rVSV-MARV is a similarly-designed surrogate virus that includes the MARV GP and a fluorescent protein reporter, mNeonGreen (mNG) fused to the VSV phosphoprotein in the genome [50]. Although MR191 has potent in vivo protective properties, it is a modest neutralizer against MARV and we determined an IC_50_ of 9.7 nM against rVSV-MARV (**Fig 3**).

DV_MR191-A878 had no activity against rVSV-EBOV or rVSV-SUDV, indicating that incorporation of MR191 Fvs as a fusion to the A878 Fvs blocked activity. However, the anti-rVSV-MARV activity of DV_MR191-A878 was similar to that of the parental MR191 (IC_50_ of 17 vs. 45 nM); but the oppositely oriented DVD-Ig with the “inner” and “outer” domains switched (DV_A878-MR191) had neutralizing activity against rVSV-EBOV (2.5 nM) and rVSV-SUDV (4.0 nM), albeit with diminished potency relative to the parental A878. Thus, there are complex orientation requirements in DVD-Igs bearing A878 and MR191 Fvs for maintaining dual activities.

Other designs incorporating MR191 and A878 Fvs had varied activity. Both AS_A878-MR191 and hSC_MR191-A878 were able to neutralize rVSV-EBOV; however, AS_A878-MR191 was ~13 fold less potent than the parental A878 mAb while hSC_MR191-A878 neutralized similarly. The scFv-Ig designs (SC_A878-MR191 and SC_MR191-A878) both had less potent activity against rVSV-EBOV than the parent (IC_50_ of 1.5 nM and 2.5 nM). SC_MR191-A774 was also tested against rVSV-SUDV and found to be similar in potency to the A774 parental (7.0 vs. 5.0 nM).

Several designs combining the Fvs of MR191 and A774 were also explored. hSC_MR191-A774 had activity against both rVSV-EBOV (4.7 nM) and rVSV-SUDV (6.8 nM), as well as rVSV-MARV (28 nM), in all cases on-par with the parental mAbs. However, the “switched” domain organization of this bsAb (hSC_A774-MR191) had lower activity against rVSV-EBOV (18 nM)). scFv-Ig versions also had lower activity against rVSV-EBOV (although, for SC_MR191-A774 it was comparable to parental A774). SC_MR191-A774 also had A774-like activity against rVSV-SUDV (7.0 nM).

### Neutralizing activity against authentic (BSL4) viruses

**Fig 4:**
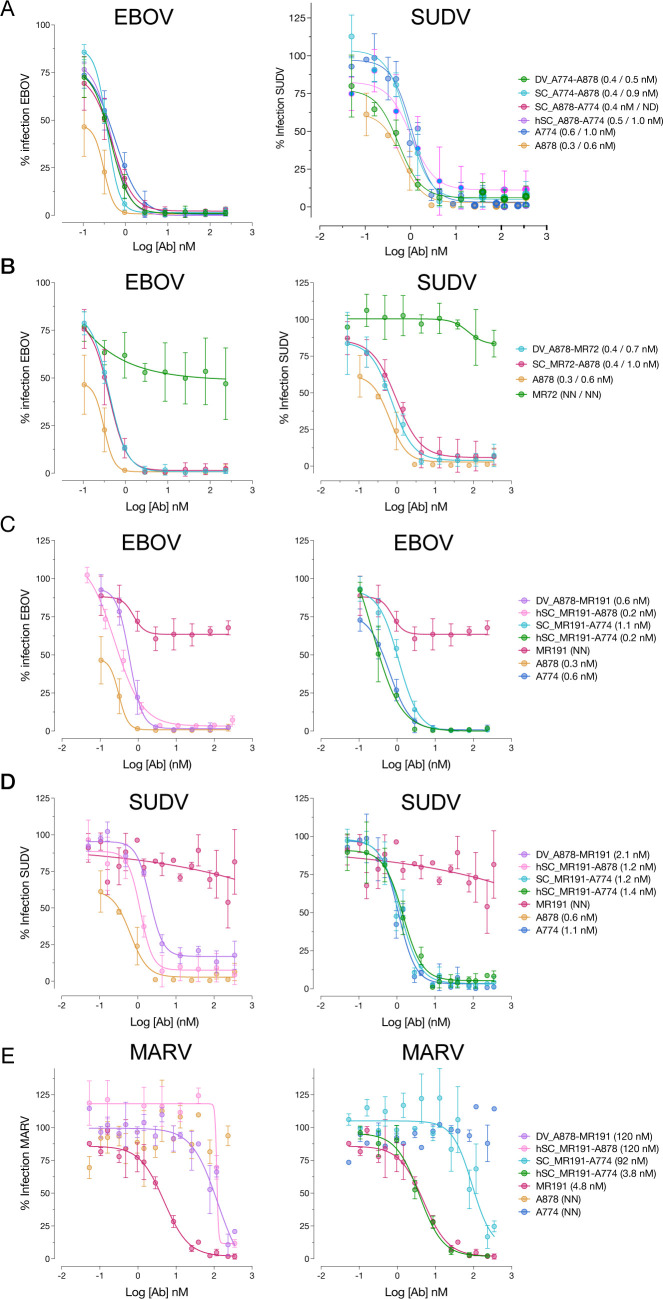
bsAbs exhibit neutralization breadth against authentic filoviruses. Neutralization curves for (A) Group I and (B) Group II bsAbs against EBOV and SUDV (respective IC_50_ values indicated in the legend in parentheses). Group III bsAb neutralization against (C) EBOV (D) SUDV and (E) MARV (IC_50_ values again in parentheses in the legend).

We tested the neutralizing activity of the most potent bsAbs from each group for activity against pathogenic ebolaviruses under BSL4 conditions using a previously described microneutralization assay (**Fig 4**) [28]. Group I bsAbs DV_A774-A878, SC_A774-A878, and hSC_A774-A878 exhibited strong (sub-nanomolar IC_50_) neutralizing activity against both EBOV and SUDV. Similarly, Group II bsAbs SC_MR72-A878 and DV_A878-MR72 strongly neutralized EBOV and SUDV. Group III bsAbs DV_A878-MR191 and SC_MR191-A774 had sub-nanomolar neutralizing activity against EBOV and SUDV, and mid-nanomolar activity against MARV (8.4 and 5.5 nM, respectively). Notably, MR191 was potently neutralizing under these conditions (0.5 nM), and hSC_MR191-A774 matched this activity against MARV while maintaining subnanomolar potency against EBOV and SUDV.

### Studies with viral escape mutants

**Fig 5:**
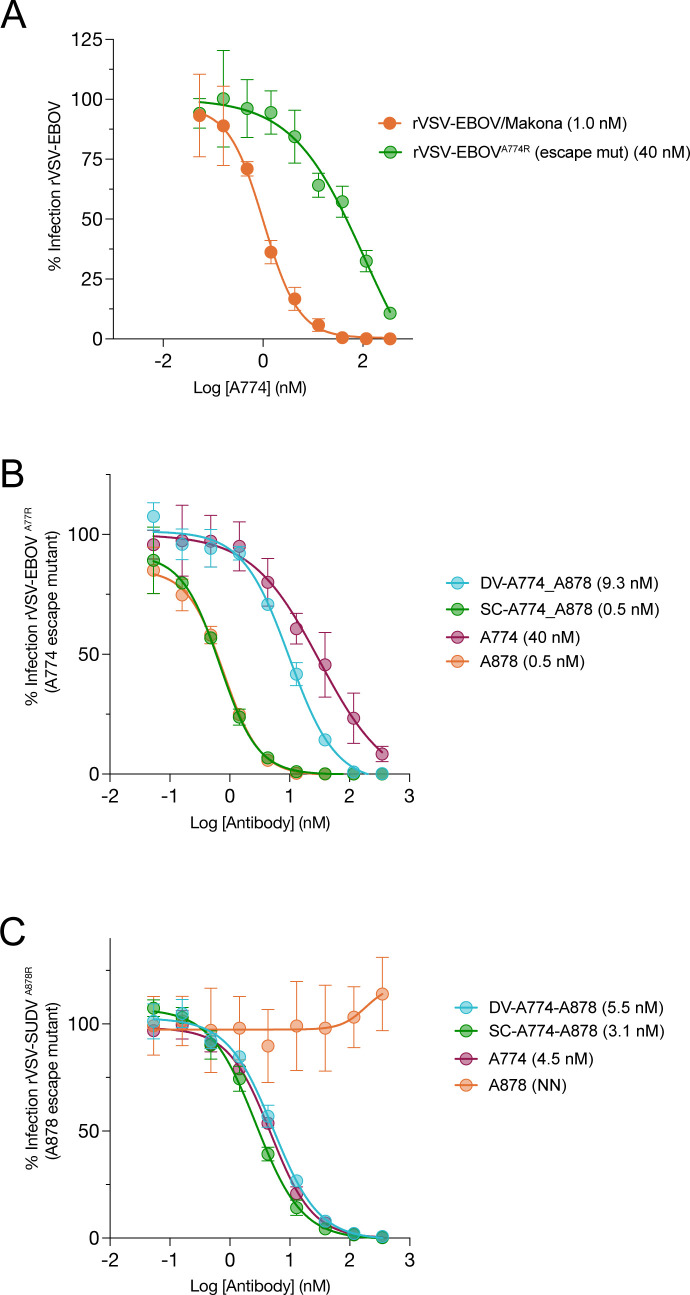
Neutralization of viral escape mutants. (A) Neutralization capacity of A774 against rVSVs bearing either WT EBOV/Makona GP or a partial neutralization escape variant GP (A774R). (B) Capacity of Group I bsAbs to neutralize VSV-EBOV^A774R^ bearing the A774 escape variant GP. (C) Capacity of Group I bsAbs to neutralize VSV-SUDV^A878R^ bearing the A878 escape variant GP. IC_50_ values indicated in the legend in parentheses.

A major advantage of targeting multiple epitopes simultaneously with a cocktail of mAbs or bsAb for viral immunotherapy is the lower risk of viral escape from the therapy by a single point mutation. To explore the potential of bsAbs to mitigate against viral escape, we separately selected escape mutants against A774 (A774R for resistant) on a rVSV-EBOV background (rVSV-EBOV^A774R^) and against A878 on an rVSV-SUDV background (rVSV-SUDV^A878R^) by viral passaging in the presence of increasing mAb. While rVSV-EBOV^A774R^ was not a complete escape, the activity of A774 against it was ~10-fold reduced (40 nM) in comparison to neutralizing activity against rVSV-EBOV (**Fig 5A and 5B**). VSV-SUDV^A878R^ was completely resistant to neutralization by A878 up to 50 nM mAb (**Fig 5C**).

BsAbs SC_A774-A878 and DV_A774-A878 were tested against both escape mutants. Both bsAbs had potent neutralizing activity against both viruses, as good as or better than the WT parental mAb. These results demonstrate that both sets of Fvs of the bsAb retained activity. Interestingly, SC_A774-A878 was even more potent against rVSV-SUDV^A878R^ than A774 (0.8 vs. 4.3 nM, respectively).

Finally, we sought to explore whether bsAbs provided resistance to viral escape. We passaged rVSV-EBOV three times (P1-P3) in the presence of SC_A774-A878 or hSC_A774-A878 (1xIC_50_ for P1, 2x for P2, and 4x for P3) but did not observe any viral escape from neutralization in the P1-P3 population (**S2 Fig**). Similar studies were attempted with DV_A774-A878 but P2 and P3 cells had unusual growth characteristics and thus the populations were not further investigated.

### Binding studies by biolayer interferometry (BLI)

**Fig 6:**
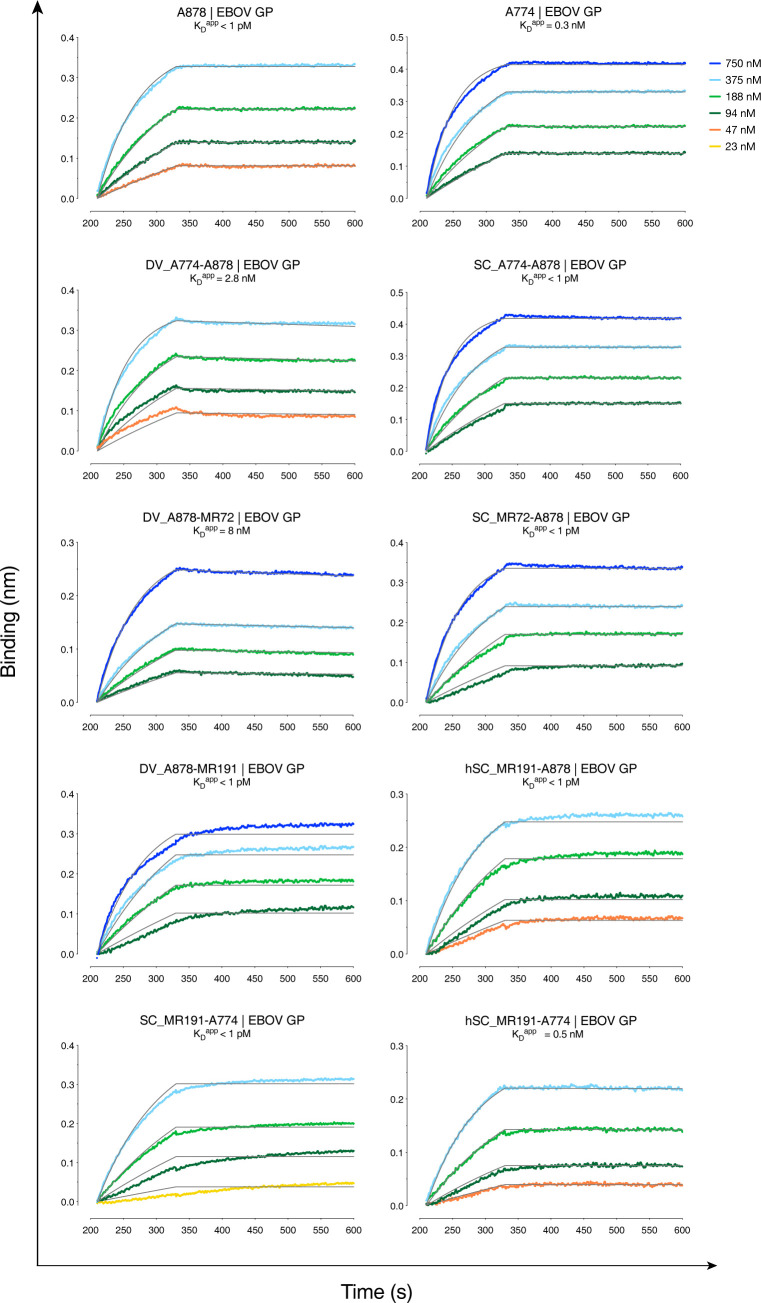
Binding profiles of bsAbs to EBOV GP and MARV GP by biolayer interferometry (BLI). Sensors were loaded with antibodies then dipped into solutions with indicated concentrations of EBOV GP. Gray lines show curve fits to a 1:1 binding model.

We measured the capacity of Group I bsAb DV_A774-A878, Group II bsAb DV_878-MR72, and Group III bsAbs hSC_MR191-A774, hSC_MR191-A878, and SC_MR191-A774 to bind EBOV GP in kinetic experiments. All five bsAbs displayed high binding affinity (**Fig 6** and **S2 Table**), with k_on_ values ranging from 2.2–6.0 x 10^4^ Ms^-1^ and slow k_off_ (1.7 x 10–4 s^-1^ and lower). Apparent dissociation constants (K_D_^app^) were in the nanomolar range for DV_774-A878 and DV_A878-MR72, and subnanomolar for hSC_MR191-A774, hSC_MR191-A878, and SC_MR191-774. As a note, given the multiple valency of the bsAbs, with four (Group I and II) or two (Group II) EBOV GP-specific binding sites per molecule, and the trimeric nature of EBOV GP, we cannot definitively determine true 1:1 binding constants with this BLI format. However, we have found the K_D_^app^ in this and other cases to be useful for comparative purposes [28,33]. Furthermore, for Group I and II bsAbs, these binding experiments do not establish that affinity of either set of Fvs is unaffected by bsAb design. Nonetheless, the results shown here demonstrate that bsAbs bind EBOV GP tightly.

**Fig 7:**
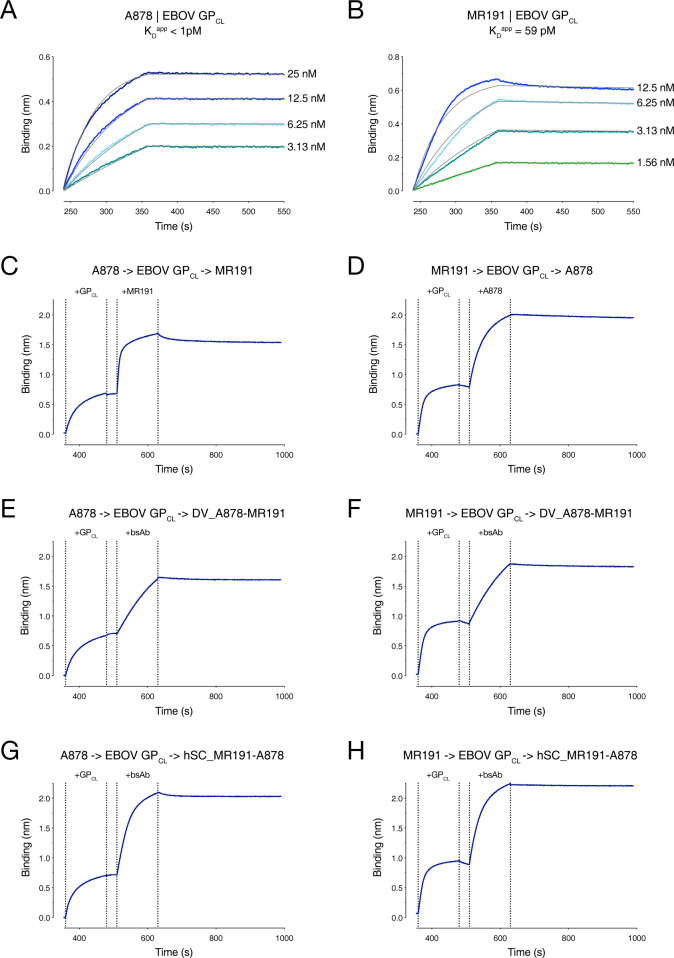
Two-phase binding of mAbs and bsAbs to EBOV GP_CL_. Kinetic binding curves for parental mAbs (**A**) A878 and (**B**) MR191 against cleaved EBOV GP (GP_CL_) were determined by BLI. Sensors loaded with mAb were then dipped into solutions with indicated concentrations of EBOV GP_CL_. (**C-H)** Two phase binding experiments for parental mAbs and group III bsAbs. Sensors were loaded with parental mAb A878 (**C,E,G**) or MR191 (**D,F,H**), then sequentially dipped into an analyte containing EBOV GP_CL_ followed by a second antibody: (**C**) MR191; (**D**) A878; (**E,F**) DV_A878-MR191; or (**G,H**) hSC_MR191-A878.

To further examine if both sets of Fvs within single molecules were active in Group III bsAbs DV_A878-MR191 and hSC_MR191-A878, we performed two-step binding experiments with “cleaved” EBOV GP (GP_CL_) (**Fig 7**). MR191 was isolated from a MARV survivor but binds to the highly conserved RBD in all filovirus GP_CL_ proteins examined to date [47]. Two-phase binding experiments with parental mAbs A878 and MR191 demonstrated that both mAbs can bind GP_CL_ simultaneously, and that the order of mAb addition does not matter. This result was expected, since the A878 epitope lies at the base of the GP, away from the RBD. Next, either the A878 parent was bound first to GP_CL_, and then each of the Group III bsAbs was added to determine if the MR191 epitope was still available. In both cases, the bsAb was able to engage the A878-GP_CL_ complex, thereby demonstrating that the MR191 Fvs on the bsAbs retains activity. The reciprocal experiment was also performed (binding of MR191 parental mAb first, followed by bsAb) with similar results. These findings show that both sets of Fvs in the two Group III bsAbs are active and bind strongly to their respective epitopes and that single bsAb molecules can recognize both of their epitopes.

### In vivo protection of pan-ebolavirus bsAbs against EBOV

**Fig 8:**
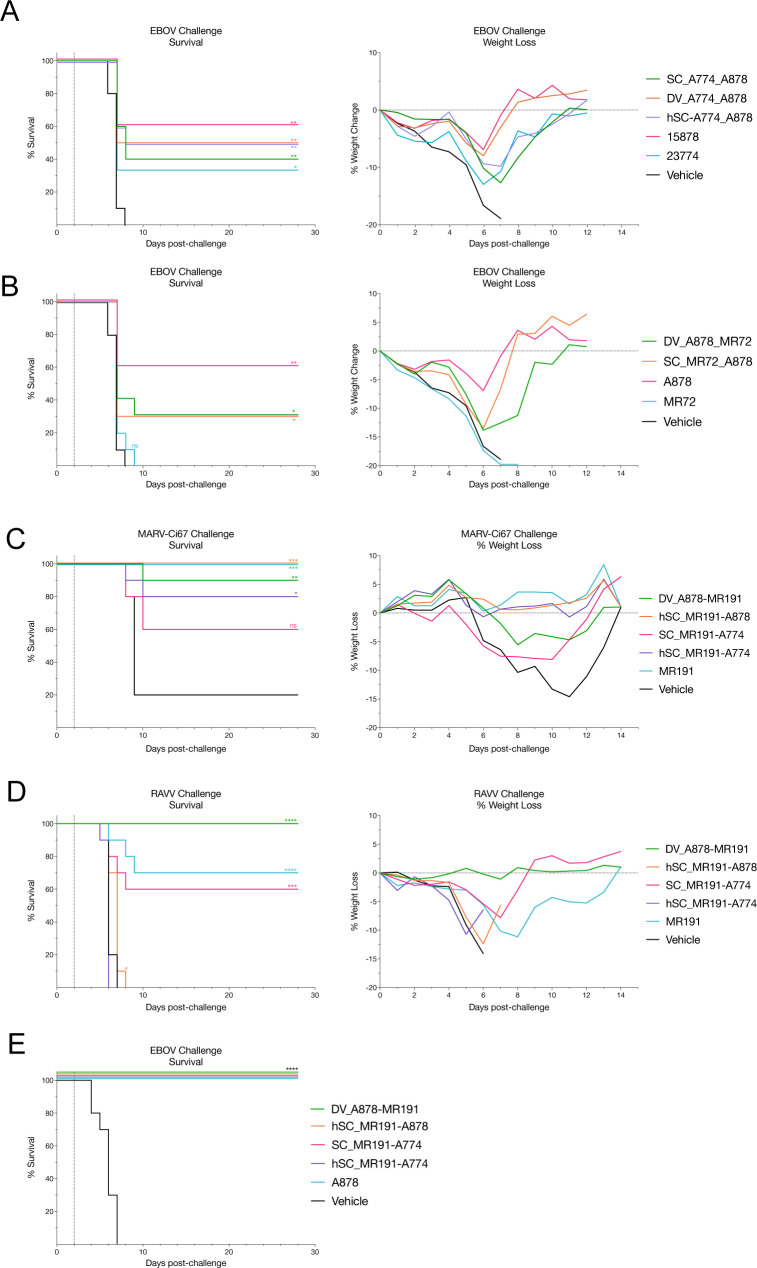
BsAbs afford broad protection against lethal challenge of divergent filoviruses in murine models. BALB/c mice challenged with 100 plaque forming units (pfu) of mouse-adapted EBOV and treated with a single dose of parental mAb A878 or A774 [100ug, ~5 mg per kilogram (mg/kg)]; bsAb [133ug, ~6.65 mg/kg, adjusted for molecular weight]; or vehicle (phosphate buffered saline, PBS) 2 days post-infection (dpi). Survival and weight loss for Group I bsAbs (**A**), Group II bsAbs (**B**), and Group III bsAbs (**E**). Survival and weight loss for type 1 interferon α/β receptor knockout (IFNAR^-/-^) mice treated with a single dose of parental mAb MR191 [100ug, ~5 mg/kg]; Group III bsAb [133ug, ~6.65 mg/kg, adjusted for molecular weight]; or vehicle (PBS) 2 dpi with 1000 pfu of MARV-Ci67 (MARV) (**C**) or Ravn virus (RAVV) (**D**). Each treatment group consisted of 10 mice. Survival curves for each group were compared to the PBS-treated group using the Log-rank (Mantel-Cox) test. (*p<0.05; **p<0.01; ***p<0.001; ****p<0.0001).

The protective efficacy of the most potent and broad bsAbs from Groups I and II were assessed in a post-exposure EBOV mouse model with conditions that resulted in partial protection by both parental mAbs A878 and A774. Following a lethal challenge with mouse adapted EBOV (EBOV-ma), mice were treated 2 days post infection (dpi) with either parental mAb, a weight-adjusted (molar) equivalent dose of bsAb, or vehicle (**Fig 8A and 8B**). Group 1 bsAbs (SC_A774-A878, DV_A774-A878, and hSC_A774-A878) exhibited a level of protective efficacy (50–60% survival) bracketed by the parental mAbs A878 (60%) and A774 (30%). Group II bsAbs DV_A878-MR72 and SC_MR72-A878 both afforded 30% survival. As expected, all mice receiving vehicle and MR72 parental mAb succumbed to infection by the 9th day. Therefore, all bsAbs retained their parent-like ability to protect against lethal EBOV infection in mice under dosing conditions in which A878 and A774 were partially protective. However, no synergistic advantage was observed in linking the two Fvs.

### Protective efficacy of Group III bsAbs protect against EBOV and MARV

To explore pan-filovirus protective potential, the most potent Group III bsAbs were tested for efficacy against lethal challenges by two different marburgviruses, Marburg virus (MARV) isolate Ci67 (**Fig 8C**) or Ravn virus (RAVV) (**Fig 8D**), in interferon–α/β-receptor1 knockout (IFNAR1^-/-^) mice [51]. Treatment with either MR191 or bsAbs was highly protective against MARV Ci67 challenge. Moreover, mice treated with MR191, hSC_MR191-A878, or hSC_MR191-A774 exhibited minimal weight loss following viral challenge. The parental mAb MR191 was also highly (70%) protective against a lethal RAVV challenge, whereas group III bsAbs varied in their protective efficacies. bsAb SC_MR191-A774 protected mice to a similar level (60%) as compared to MR191. Interestingly, DV_A878-MR191 afforded complete protection (100%) with no observable weight loss. However, Group III hSC-format bsAbs afforded less protection against RAVV challenge—only hSC_MR191-A878 delayed death by a day compared to vehicle-treated mice. Although more work is needed, one possibility is that these differences are due to differences in the *in vivo* half lives of the antibodies.

BsAbs hSC_MR191-A878 and DV_A878-MR191 were also tested for protective efficacy against EBOV (**Fig 8E**). Both bsAbs conferred 100% protection against EBOV as did A878, whereas vehicle-treated mice succumbed by day 7. Interestingly, DV_A878-MR191 could completely protect mice despite its lower neutralization potency against EBOV.

## Discussion

Here we explore the potential of bsAbs for broad neutralization and protection against filoviruses. In Group I bsAbs, we combined the Fvs of the MBP134 cocktail in various configurations with the goal of identifying pan-ebolavirus neutralizing bsAbs. We found that many formats retained A878-like broad neutralizing potency, although a few constructs (DV_A774-A878, DV_A878-A774) had reduced neutralizing activity. However, this activity was still on-par or stronger than the weaker of the two parental mAbs (A774). The cause for this lower potency is unclear at present, but may be due to suboptimal VH-VL interactions or inaccessibility of one of the sets of Fvs when incorporated into the DVD-Ig format. DV_A774-A878 nonetheless had strong binding to EBOV GP, as assessed by BLI. Further, asymmetric bsAb AS_A774-878 was also less potent than A878, possibly due to the lower avidity of the asymmetric Duobody format, which contains only one copy of each set of Fvs relative to DVD-Ig, scFv-Ig, or Bis4 formats, which contain two copies of each set of Fvs. Several Group I bsAbs performed similarly to parental mAbs A878 and A774 under EBOV challenge conditions where they were partially protective, but no distinctive advantage to the bsAbs in terms of efficacy was observed. Importantly however, two of the Group I bsAbs (DV_A772-A878, SC_A774-A878) retained neutralizing activity against rVSV-EBOV^A774R^ and rVSV-SUDV^A878R^. Additionally, neutralizing activities of SC_A774-A878 or hSC_A774-A878 were impervious to development of viral escape upon passaging. There is thus an advantage of the bsAbs relative to monospecific mAb parents in lowering susceptibility to antibody resistance by mutation.

Group II bsAbs were designed with a “Trojan Horse” mechanism in mind [36,37]. Since RBS-targeting MR72 is poorly neutralizing against ebolaviruses, the degree to which A878 or A774 Fvs retained their potency could be more easily assessed. All Group II bsAbs except SC_A774-MR72 neutralized rVSV-EBOV and, in those cases tested, rVSV-SUDV, as potently as the parental neutralizing mAb. The cause for lower potency of SC_A774-MR72 is not clear. Group II bsAbs that were tested for protective efficacy were also partially protective (30%) under conditions where A878 and A774 were also partially protective. Parental mAb MR72 was not protective under these dosing conditions, likely because the RBS is not accessible in the prefusion GP. Thus, the partial protective activity of Group II bsAbs is due solely to the A878 Fvs, but no advantage was conferred by physical linkage of these Fvs to MR72. By contrast, earlier Trojan Horse bsAbs were ’obligate bispecifics’ that were completely reliant on both sets of Fvs, one for broad viral recognition (FVM09) and the other for inhibition of RBS-receptor interactions in endosomes (MR72) [36].

To test if a single bsAb could afford pan-filovirus protection, Group III bsAbs were engineered to combine Fvs from the MARV-protective mAb MR191 with those from A878 or A774. Of these molecules, only hSC_MR191-A878 and AS_A878-MR191 had A878-like potency against rVSV-EBOV and rVSV-SUDV. For the asymmetric bsAb, this retention of activity contrasts with that of the Group I asymmetric molecule but is consistent with the Group II asymmetric molecule. Thus, the A878 Fvs may be equally potent as a single “arm” if paired with certain Fvs. All other Group III bsAbs had lower (but still strong) potency against rVSV-EBOV and, in cases tested, rVSV-SUDV. The Group III bsAbs all exhibited moderate neutralizing activity against rVSV-MARV, consistent with the parental MR191 mAb. Several Group III bsAbs, however, exhibited strong protection against MARV-Ci67, and DV_A878-MR191 afforded complete protection against RAVV. Interestingly hSC_MR191-A774 was not protective against either MARV challenge strain. All Group III bsAbs tested retained their ability to protect against EBOV challenge under conditions where A878 was fully protective. These results demonstrate that linkage of Fvs from pan-ebolavirus mAb ADI-15878 with those of MARV-protective mAb MR191 affords pan-filovirus protection *in vivo*.

No neutralizing or protective synergy was detected in any of the pan-ebolavirus bsAb constructs tested here, relative to their parental mAbs. We and others have previously reported synergistic activity of bsAbs in other systems. For example, a DVD-Ig containing Fvs from two Crimean Congo Hemorrhagic Fever virus (CCHFV) mAbs (DVD-121-801) was protective against lethal challenge with a therapeutic dosing regimen, whereas a cocktail of the parental mAbs (ADI-36121 and ADI-36801) was not [34]. Moreover, improved neutralization profiles and breadth were observed with this and other DVD-Igs relative to the cocktails. An asymmetric HIV-1 bsAb consisting of one virus-targeting arm (10E8v2.0) and one host receptor-targeting arm (iMab) had much greater breadth and neutralizing potency than parental mAbs and reduced HIV-1 viral load in humanized mice more potently than either parental mAb alone [40]. However, the precise mechanism for the synergistic neutralization and/or protective properties of bsAbs is difficult to design or predict *a priori*. In the case of pan-filovirus bsAbs, given the differences in domain topology and accessibility of the base epitope on ebolavirus GP and the RBS on MARV GP, the bsAbs we describe herein are the only purely virus-specific molecules with cross-clade protective activity described to date.

Given the capacity of bsAbs to target multiple epitopes simultaneously, a potentially key benefit is decreased susceptibility to viral escape, demonstrated here with DV_A774-A878 and SC_A774-A878 neutralization of rVSV-EBOV^A774R^ and rVSV-SUDV^A878R^ and passaging studies with SC_A774-A878 or hSC_A774-A878. Similarly, a trispecific HIV-1 mAb (tsAb) was more effective at suppressing viral rebound in non-human primates challenged with a mixture of viruses than parental mAbs [52]. Such multiepitope targeting likely underlies the increase in breadth observed with HIV-1 bsAbs as well. Although a similar effect could be obtained with a cocktail of mAbs, the bsAbs have the theoretical advantage of more facile production, since only a single molecule must be expressed.

While many bsAb scaffolds are available for engineering, each with their own advantages and disadvantages, we have found here and in other cases that the selection of formats for optimal activity often relies on empirical testing. For example, the DVD-Ig platform does not contain long Gly/Ser polypeptide linker regions of scFvs present in the scFv-Ig and BiS4 formats which are liabilities for proteolysis, but in some cases the activity of the “inner” set of Fvs can be blocked. This is the likely cause for the lack of neutralizing activity against rVSV-EBOV observed for DV_MR191-A878 and DV_MR191-A774, and may explain the lower neutralizing activity of DV_A774-A878 against the A774 resistant virus (rVSV-EBOV^A774R^) relative to parental A878. However, in other cases (e.g., “Trojan horse” bsAbs targeting host and viral components), we have found that the activity of the inner Fvs is unaffected in the DVD-Ig format [36]. Similarly, “asymmetric” formats are more “IgG-like” and potentially may be less immunogenic than other “tetravalent” formats, but also contain less avidity for each set of Fvs. In the context of the viral surface, this may result in lower activity relative to formats where there are two copies of each set of Fvs as we have reported here. Thus, there is a benefit to exploring multiple formats and Fv pairs for bsAb design.

BsAbs continue to be a promising platform for development of novel immunotherapeutics for filoviruses and other pathogens. Here we examine the requirements for broad and potent neutralizing activity in bsAbs constructed from MBP134 constituents, as well as the only bsAbs with pan-filovirus neutralizing and protective activity. These results provide insights into design rules for bsAbs and yield novel candidates for downstream development.

## Materials and methods

### Ethics statement

Animal protocols were conducted under institutional animal care and use committee (IACUC)-approved protocols in compliance with the Animal Welfare Act, Public Health Service Policy, and other applicable federal statutes and regulations relating to animals and experiments involving animals. The facilities where these studies were conducted (USAMRIID and NEIDL, Boston University) are accredited by the Association for Assessment and Accreditation of Laboratory Animal Care, International and adhere to principles stated in the Guide for the Care and Use of Laboratory Animals, National Research Council.

### Cells

Vero cells were maintained in high-glucose Dulbecco’s modified Eagle medium (DMEM; ThermoFisher) supplemented with 10% fetal bovine serum (FBS; Atlanta Biologicals), 1% GlutaMAX (Life Technologies) and 1% penicillin/streptomycin (Life Technologies) at 37°C, with 5% CO_2_ in a humidified incubator. Freestyle 293-F cells (ThermoFisher) were maintained in Freestyle 293 expression media (Thermofisher) with 1% penicillin/streptomycin (Life Technologies) at 37°C, with 8% CO_2_ in a humidified shaking incubator.

### Viruses

Generation and propagation of recombinant vesicular stomatitis viruses (rVSV) bearing the GP from either EBOV/Mayinga (EBOV/H.sap-tc/COD/76/ Yambuku-Mayinga), or SUDV/Boneface (SUDV/C.por-lab/SSD/76/Boniface) in place of VSV G and encoding enhanced green fluorescent protein (eGFP) in the first position as well as an rVSV bearing GP from (MARV/H.sap-tc/KEN/80/Mt. Elgon-Musoke) and encoding an mNeongreen-phosphoprotein P (mNG-P) fusion protein was previously described [36,49,53,54].

### Antibody expression and purification

To generate Dual Variable Domain (DVD) bispecific antibodies, synthetic genes encoding the outer variable domains were linked to the N-terminus of the inner variable domains via short peptide linkers, “ASTKGP” and “TVAAP” for the heavy and light chains respectively, and subcloned into the pMAZ IgH and IgL vectors. For BiS4 bispecific antibodies, synthetic genes encoding single chain variable fragments (scFv) were inserted into the upper hinge region with flanking GGGSx2 linkers between C220 and D221 as reported previously [46]. pMAZ IgH and IgL were co-transfected into Freestyle 293-F cells (ThermoFisher) using linear polyethylenimine (Polysciences) and cultured in Freestyle expression media in a humidified shaking incubator at 37°C with 8% CO2 for 6 days. Cells were pelleted by centrifugation and the clarified supernatant was incubated with Protein A resin (1ml for 600ml supernatant) for 2 hours at 4°C. Antibodies were purified according to the manufacturer’s protocol using the Gentle antibody elution system (Thermofisher Scientific) and buffer exchanged into Hepes buffer (200mM NaCl, 150mM HEPES[pH 7.4]). Antibodies were concentrated using Amicon centrifugal filter units (Millipore Sigma) with a nominal molecular weight cutoff of 50 kDa.

### Viral escape mutation selection

A concentration of mAb corresponding to the 90% inhibitory concentration value derived from the virus neutralization curve was preincubated with a 3-fold serial dilution of VSV-EBOV/Makona GP prior to addition onto a confluent monolayer of Vero cells in duplicate. Infection was allowed to proceed until >90% of cell death (determined by eye) was achieved. Viral supernatant was collected from the well that received the least amount of viral inoculum and utilized for subsequent passages under increasing concentrations of antibody selection. Passage 3 supernatants were collected and tested for viral neutralization escape. If escape was observed then individual viral clones were plaque purified on Vero cells and sequenced to determine their GP gene sequence as described previously [49].

### rVSV neutralization

A pre-titrated amount of rVSVs bearing the GP of EBOV, SUDV, or MARV (MOI ≈ 1 infectious unit (IU) per cell) was incubated with a dilution series of antibody for 1h at room temperature, prior to addition onto vero cell monolayers. When viral particles bearing cleaved EBOV GP (GP_CL_) were utilized, viral particles were first incubated with thermolysin (200 μg/mL, pH 7.5; Sigma-Aldrich) for 1hr at 37°C. Reactions were then stopped by placing onto ice along with the addition of phosphoramidon (1 mM) as previously described [49] and immediately utilized in assay. Viral infectivity was measured by automated enumeration of eGFP^+^ or mNG^+^ cells using a Cytation 5 reader at 12–14 hours post-infection. Data were subjected to non-linear regression analysis to extract half maximal inhibitory concentration (IC_50_) values (4-parameter, variable slope sigmoidal dose-response equation; GraphPad Prism). Relative IC_50_ values were calculated for all curves with sigmoidal curves and absolute IC_50_ values were calculated for curves with ill-defined plateaus.

### Biolayer interferometry (BLI)

The antibody binding properties were determined by biolayer interferometry using the OctetRed (Fortebio, Pall LLC). Antibody was initially loaded onto anti-human Fc sensors (Sartoris) and subsequently followed by EBOV GP, GP_CL_, or MARV GP association and dissociation. Global fitting to a 1:1 binding model was used to estimate k_on_ (association rate constant), k_off_ (dissociation rate constant) and K_D_^app^ (apparent equilibrium constant). Although data could be described accurately with a 1:1 model, given the bivalent nature of the antibody, and the trivalent nature of GP, we cannot rule out avidity effects and therefore report apparent K_D_.

### Authentic filovirus neutralization assays

A dilution series of antibodies was incubated with either Ebola virus/H.sapienstc/COD/1995/Kikwit-9510621 (EBOV/Kik-9510621; ‘EBOV-Zaire 1995’), Sudan virus/H. sapiens-gp-tc/SDN/1976/BonifaceUSAMRIID111808 (SUDV/Bon-USAMRIID111808; ‘SUDV-Boniface 1976’), or Marburg virus/*H*.*sapiens*tc/DEU/1967/Hesse-Ci67 for 1h at 37°C and then incubated on Vero E6 cells for another 1h at 37°C. Antibody/virus inoculum was removed and fresh media added. At 48h post infection, cells were fixed and blocked with 1% bovine serum albumin. Cells were immunostained for EBOV, SUDV or MARV infection with either EBOV specific mAb KZ52, SUDV GP-specific mAb 3C10, or MARV GP-specific mAb 9G4, respectively. Cells were washed with PBS prior to incubation and either goat anti-human IgG or goat anti-mouse IgG conjugated to Alexa Fluor 488 (Invitrogen) and subsequent counterstaining with Hoescht stain (Invitrogen). Quantitation of infected cells was determined by fluorescence microscopy and automated image analysis using an Operetta high content device (Perkin Elmer) and the image analysis Harmony software, as previously described [36].

### Ebola virus mouse study

Groups of 10, 8–12 week old female BALB/c mice were intraperitoneally (I.P.) inoculated with 100 plaque forming units (PFU) of Mouse-adapted EBOV/Mayinga (EBOV-ma)(EBOV/M.mus-tc/COD/76/Yambuku-Mayinga) [55]. Day 2 post infection, mice were treated via I.P. route with either vehicle, 133 ug bsAb (dose adjusted to account for the higher molecular weight of the bsAb), or 100 ug of mAb. Mice were observed daily for 28 days for moribund condition. Moribund mice were euthanized when euthanasia criteria was met according to IACUC approved protocol.

### Marburg mouse study

Groups of 10, 5–8 week old male and female Interferon-alpha/beta receptor knockout (IFNAR -/-) mice were intraperitoneally (I.P.) inoculated with 1000 PFU of MARV-Ci67 or RAVN. On day 2 post infection, mice were treated via I.P. route with either vehicle, 133ug bsAb, or 100ug of mAb. Mice were weighed in groups and observed daily for moribund condition for 28 days. Moribund mice were euthanized when euthanasia criteria was met according to IACUC approved protocol.

## Supporting information

S1 Table bsAb Design. (PDF)

S2 Table Biolayer Interferometry for binding EBOV GP. (PDF)

S1 Fig Neutralization studies agianst rVSV-SUDV for A061 Fv-containing bsAbs. IC_50_ values indicated in the parentheses in the legend.
(PDF)

S2 Fig Passage of rVSV-EBOV against bsAbs. Neutralization of viral populations from three passages (P1-P3) for bsAbs.
(PDF)
