# Supplementary material for: Design and characterization of protective pan-ebolavirus and pan-filovirus bispecific antibodies
Source: PLoS Pathog. 2024 Apr 11;20(4):e1012134. doi: 10.1371/journal.ppat.1012134 (PMC11037526; doi:10.1371/journal.ppat.1012134)
Supplement: S1 Table — (PDF) [file ppat.1012134.s001.pdf]

Table S1 – bsAb Design				
**Name**	**Group**	**Format**	**Added Fv (Ab2)**	**Parental (Ab1)**
DV_A774-A878	I	DVD-IgG	ADI-23774	ADI-15878
SC_A774-A878	I	C' scFv-IgG	ADI-23774	ADI-15878
hSC_A774-A878	I	hinge scFv-IgG	ADI-23774	ADI-15878
AS_A774-A878	I	Duobody	ADI-23774	ADI-15878
DV_A878-A774	I	DVD-IgG	ADI-15878	ADI-23774
SC_A878-A774	I	C' scFv-IgG	ADI-15878	ADI-23774
hSC_A878-A774	I	hinge scFv-IgG	ADI-15878	ADI-23774
DV_A878-A061	I	DVD-IgG	ADI-15878	ADI-16061
DV_A061-A774	I	DVD-IgG	ADI-16061	ADI-23774
SC_A878-MR72	II	C' scFv-IgG	ADI-15878	MR72
DV_A878-MR72	II	DVD-IgG	ADI-15878	MR72
SC_MR72-A878	II	C' scFv-IgG	MR72	ADI-15878
AS_A878-MR72	II	Duobody	ADI-15878	MR72
AS_A774-MR72	II	Duobody	ADI-23774	MR72
SC_MR72-A774	II	C' scFv-IgG	MR72	ADI-23774
SC_A774-MR72	II	C' scFv-IgG	ADI-23774	MR72
DV_A878-MR191	III	DVD-IgG	ADI-15878	MR191
SC_A878-MR191	III	C' scFv-IgG	ADI-15878	MR191
hSC_A878-MR191	III	hinge scFv-IgG	ADI-15878	MR191
DV_MR191-A878	III	DVD-IgG	MR191	ADI-15878
SC_MR191-A878	III	C' scFv-IgG	MR191	ADI-15878
hSC_MR191_A878	III	hinge scFv-IgG	MR191	ADI-15878
AS_A878-MR191	III	Duobody	ADI-15878	MR191
AS_A774-MR191	III	Duobody	ADI-23774	MR191
DV_MR191-A774	III	DVD-IgG	MR191	ADI-23774
SC_MR191-A774	III	C' scFv-IgG	MR191	ADI-23774
hSC_MR191-A774	III	hinge scFv-IgG	MR191	ADI-23774
SC_A774-MR191	III	C' scFv-IgG	ADI-23774	MR191
hSC__A774-MR191	III	hinge scFv-IgG	ADI-23774	MR191
