# Supplementary material for: Design and characterization of protective pan-ebolavirus and pan-filovirus bispecific antibodies
Source: PLoS Pathog. 2024 Apr 11;20(4):e1012134. doi: 10.1371/journal.ppat.1012134 (PMC11037526; doi:10.1371/journal.ppat.1012134)
Supplement: S2 Table — (PDF) [file ppat.1012134.s002.pdf]

Table S2 – Biolayer Interferometry for binding EBOV GP			
**Antibody**	**K_D^{app} (M)**	**k_{on} (M s)$^{-1}$**	**k_{off} (s^{-1})**
ADI-15878	$< 1.0 \times 10^{-12}$	$(4.49 \pm 0.03) \times 10^{4}$	$< 1.0 \times 10^{-7}$
ADI-23774	$(3.0 \pm 0.7) \times 10^{-10}$	$(2.72 \pm 0.02) \times 10^{4}$	$(8.2 \pm 2.0) \times 10^{-6}$
DV_A774-A878	$(2.8 \pm 0.2) \times 10^{-10}$	$(5.93 \pm 0.08) \times 10^{4}$	$(1.7 \pm 0.1) \times 10^{-4}$
SC_A774-A878	$< 1.0 \times 10^{-12}$	$(1.69 \pm 0.02) \times 10^{4}$	$< 1.0 \times 10^{-7}$
DV_A878-MR72	$(8.1 \pm 0.2) \times 10^{-9}$	$(2.27 \pm 0.02) \times 10^{4}$	$(1.8 \pm 0.1) \times 10^{-4}$
SC_MR72-A878	$< 1.0 \times 10^{-12}$	$(1.08 \pm 0.02) \times 10^{4}$	$< 1.0 \times 10^{-7}$
DV_A878-MR191	$< 1.0 \times 10^{-12}$	$(1.92 \pm 0.06) \times 10^{4}$	$< 1.0 \times 10^{-7}$
hSC_MR191-A878	$< 1.0 \times 10^{-12}$	$(3.39 \pm 0.10) \times 10^{4}$	$< 1.0 \times 10^{-7}$
SC_MR191-A774	$< 1.0 \times 10^{-12}$	$(2.80 \pm 0.10) \times 10^{4}$	$< 1.0 \times 10^{-7}$
hSC_MR191-A774	$(5.2 \pm 0.6) \times 10^{-10}$	$(3.57 \pm 0.03) \times 10^{4}$	$(1.9 \pm 0.2) \times 10^{-5}$
