# Supplementary material for: Design and characterization of protective pan-ebolavirus and pan-filovirus bispecific antibodies
Source: PLoS Pathog. 2024 Apr 11;20(4):e1012134. doi: 10.1371/journal.ppat.1012134 (PMC11037526; doi:10.1371/journal.ppat.1012134)
Supplement: S1 Fig — IC50 values indicated in the parentheses in the legend. (PDF) [file ppat.1012134.s003.pdf]

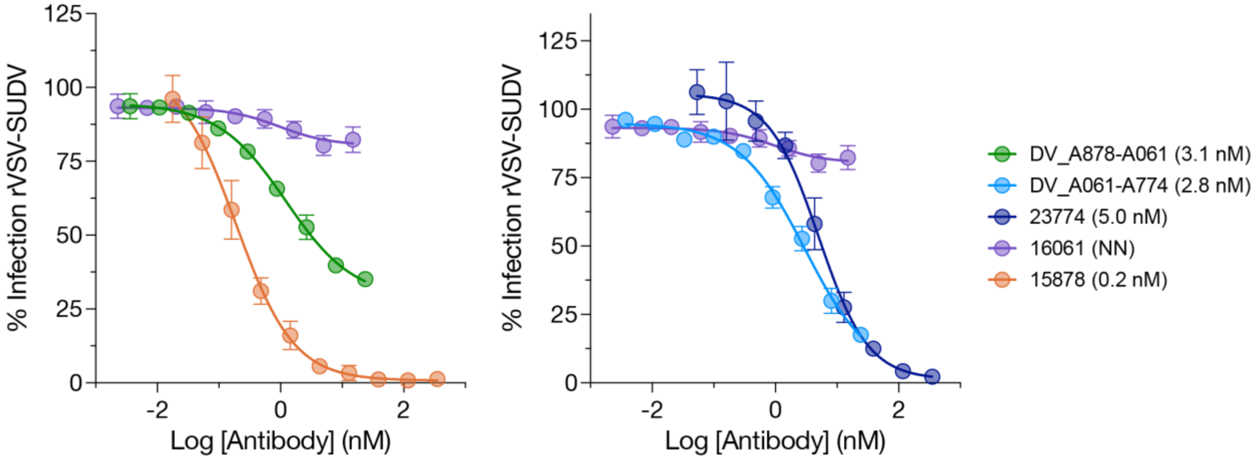

Figure S1. Neutralization studies agianst rVSV-SUDV for A061 Fv-containing bsAbs. IC_{50} values indicated in the parentheses in the legend.
