# Supplementary material for: Design and characterization of protective pan-ebolavirus and pan-filovirus bispecific antibodies
Source: PLoS Pathog. 2024 Apr 11;20(4):e1012134. doi: 10.1371/journal.ppat.1012134 (PMC11037526; doi:10.1371/journal.ppat.1012134)
Supplement: S2 Fig — Neutralization of viral populations from three passages (P1-P3) for bsAbs. (PDF) [file ppat.1012134.s004.pdf]

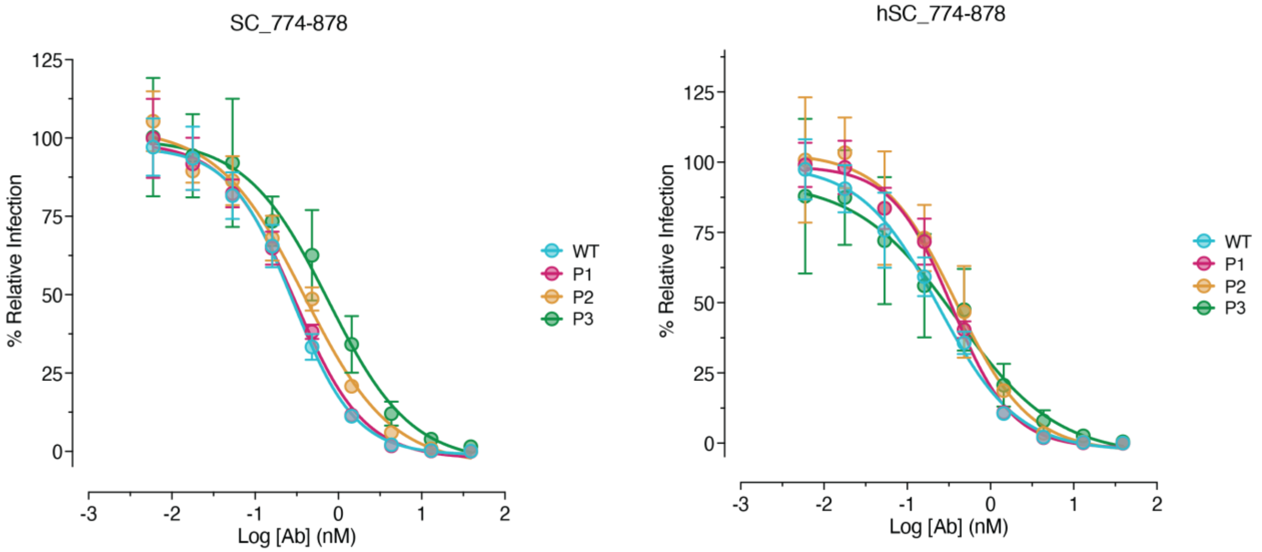

Figure S2. Passage of rVSV-EBOV against bsAbs. Neutralization of viral populations from three passages (P1-P3) for bsAbs.
